# Everybody's Page

**Published:** 1888-09-01

**Authors:** 


### Everybody's Page.

One out of about 3·3 deaths is due to diseases affecting the respiratory organs.

Milk is frequently skimmed and then mixed with fresh milk, and sold as whole milk.

Long-continued exposure to bright light is to be guarded against; those who travel in snowy regions ought to wear preservers.

It has been estimated that if the heat generated within the body were allowed to accumulate within us, and none to be given off, it would be sufficient to raise the body to the boiling point in thirty-six hours.

Scientists tell us that at an early period in the history of the universe what is now the sun had not its present form, but that its elements existed as a diffused nebular mass. At that period there was only diffuse light, but, as development proceeded this nebular mass became condensed into what we now know as the sun, which ministers so much to the light and life of our planet.

In Brazil there is a tribe called the Cafusos which has sprung into existence by crossing between the long, stiff-haired natives and the imported negro slaves. As might naturally be expected from the admixture of these extremes, this people possess hair of a very extraordinary kind. It rises perpendicularly from the head in close curly masses, and forms a wig of such enormous dimensions that the possessors must stoop low when entering their huts.

Many of the microscopic organisms that produce disease not only do not flourish in sunlight, but seem actually to be destroyed by it. Tyndall found that flasks containing Pasteur's solution (a fluid favourable to the development of lower organisms) which had been exposed to the sun remained more free of germs than those placed in the shade. These experiments have been confirmed by others, and recently Duclaux has shown that sunlight is fatal to those organisms which we consider to be unfavourable to the healthy existence of the human body.

The winter of 1880 was marked by dense fogs. During that time it was noted in the Zoological Gardens "that the health of the animals was seriously affected by the darkness, plants under glass in the Botanical Gardens ceased to grow, and showed evidence of seriously-depressed vitality during this period of darkness, from the absence of the chemical influence of the sun's rays." The death-rate in London was raised by forty per cent., almost exclusively on account of respiratory complaints, an increase equal to that caused by a cholera epidemic; but because the increase was not due to any of the epidemic diseases but only smoke and fog, it did not excite in the minds of the public any serious alarm.

Cats have always been ornamental rather than useful, and the more fortunate of them lead the lives of refined hedonists. It was a stroke of genius to find an occupation in life for the laziest exquisites among the domestic animals. The happy inspiration came to a gentleman of Chicago, who had reason to suspect that his house drains were defective. He threw the entire establishment open to the inspection of his cat, and then poured a strong infusion of valerian into the pipes. The plot succeeded to a marvel. Puss, who is as fond of perfumes as any client of Mr. Rimmel's, soon discovered three leaks, at each of which she established herself in turn, to enjoy the pungent odour at her leisure. Thus, not only were the householder's suspicions confirmed, but the actual situation of the leaks was revealed as accurately as if they had been indicated by a sanitary inspector.

Both silk and felt hats, by their stiff, hard margins, exercise a constricting influence around the head and compress both the blood-vessels and nerves of the scalp. In addition to this, they are seldom provided with proper means for ventilation, and therefore prevent that free evaporation from the scalp which is so essential for the health of the hair and its follicle.

Ferrier has, by patient inquiry, mapped out the various areas which he holds to be centres from which, in the phenomena of voluntary movements, influences pass to special groups of muscles; and he has continued his investigations in the direction of destroying these centres, and has found that paralysis of the muscles which contract on the irritation of electricity, is the result.

If we examine the statistics of the last decade, 1871-81, when sanitary measures have begun to bear fruit, we find that the death-rate has fallen about 4½ per cent. in England and Wales, viz., from 22·5 to 21·27; while the lower death-rate of this decade, says the Registrar-General, "implies the survival of 299,385 persons, who, with the previous rate of mortality, would have died."

A very simple little instrument for the examination of milk by colour has recently been invented in Germany—Heeren patent milk tester. It is made of vulcanite, and on the raised portion of a round disc a little milk is placed; over this there is put a glass cover, which spreads out the milk. Round the glass is painted various shades of colour representing cream, very fat milk, normal, less fat, poor, and very poor.

Dr. Vibert, who is connected with the Paris Morgue, has stated that among two hundred post-mortem examinations which he made on persons who died a violent death, he has in as many as twenty per cent. found evidence of old tubercular lesions, which had healed. Such evidence as this goes far to prove that consumption is not the hopelessly incurable disease it is usually considered, and gives hope that the best results may be obtained by early and careful treatment.

No sooner does a cold blast impinge upon us than the skin, richly provided with blood-vessels and nerves, contracts, and the surface is often roughened with little points in consequence of this contraction. In thus contracting its blood-vessels become emptied of their blood, and its heat-radiating and evaporating function is for the time arrested, while the body goes on accumulating heat sufficient to furnish a reaction when the cold is withdrawn, or before it. This reaction or glow is one of the best indications that we have not been injured by the cold.

The electric light produces, as is well known, disturbance of vision among the workmen who are exposed to it. Recently a new pathological result of working in intense light has been reported to the Paris Surgical Society. The cases were those of workmen at Creusot, where an electric furnace is used for quickly melting metals. The men suffer greatly from the effects of the intense light, which exceeds a hundred thousand candle power. After one or two hours the workers have a painful sensation in the throat, face, and temples, while the skin becomes copper-red in hue, and an eye-irritation lasts forty-eight hours, the discharge of tears being copious. After five days the skin peels off. All these effects are produced by light alone, no heat being felt. Dark-coloured glass mitigates the effects somewhat, but does not entirely prevent them.

